# Analysis of Von Kármán Swirling Flows Due to a Porous Rotating Disk Electrode

**DOI:** 10.3390/mi14030582

**Published:** 2023-02-28

**Authors:** James Visuvasam, Hammad Alotaibi

**Affiliations:** 1 Department of Mathematics, Rathnavel Subramaniam College of Arts and Science (Autonomous), Coimbatore 641 402, Tamil Nadu, India; 2 Department of Mathematics and Statistics, Faculty of Science, Taif University, P.O. Box 11099, Taif 21944, Saudi Arabia

**Keywords:** mathematical modeling, non-linear differential equations, rotating disk electrodes, homotopy analysis method

## Abstract

The study of Von Kármán swirling flow is a subject of active interest due to its applications in a wide range of fields, including biofuel manufacturing, rotating heat exchangers, rotating disc reactors, liquid metal pumping engines, food processing, electric power generating systems, designs of multi-pore distributors, and many others. This paper focusses on investigating Von Kármán swirling flows of viscous incompressible fluid due to a rotating disk electrode. The model is based on a system of four coupled second-order non-linear differential equations. The purpose of the present communication is to derive analytical expressions of velocity components by solving the non-linear equations using the homotopy analysis method. Combined effects of the slip λ and porosity γ parameters are studied in detail. If either parameter is increased, all velocity components are reduced, as both have the same effect on the mean velocity profiles. The porosity parameter γ increases the moment coefficient at the disk surface, which monotonically decreases with the slip parameter λ. The analytical results are also compared with numerical solutions, which are in satisfactory agreement. Furthermore, the effects of porosity and slip parameters on velocity profiles are discussed.

## 1. Introduction

In fluid mechanics, swirling flows are a common phenomenon, which have attracted significant attention due to their numerous industrial, mechanical, and environmental applications, such as rotating types of machinery (rotary pumps, fans, turbine, etc.), boilers, chemical storage, cyclone separators, and nuclear reactors spinning discs [1]. Rotating disk electrodes (RDE), incredibly porous and rough ones, are commonly used to measure electrochemical reactions, such as oxygen reduction reactions, catalysis, [2] and electrode kinetics [3]. Miklavcic et al. [4] studied the effect of slip parameters on various normalized slip coefficient ranges, and Turkyilmazoglu et al. [5] examined a swirling flow caused by a rotating rough and porous disk in terms of heat and mass transfer.

A disk with an infinite rotation was first described by Von Kármán in 1921 [6]. Von Kármán [6] also examined the similarity changes between partial and ordinary differential equations. Furthermore, Von Kármán formulated steady and unsteady states for this equation. Asymptotic solutions for steady-state conditions have been reported by Cochran [7], and non-steady-state solutions have been reported by Benton [8]. Millsaps and Pohlhausen [9] described the constant-temperature warmness switch values from a rotating disk using various Prandtl numbers.

Attia et al. [10,11,12] described how an external uniform magnetic field affects the flow caused by a rotating disk. Stuart [13], Kuiken [14], and Ockendon [15] investigated steady hydrodynamic flow induced by a rotating disk with the effect of uniform injection or suction. Using homotopy analysis, Rashidi et al. [16] derived approximate analytical solutions for heat transfer in porous mediums of steady flow over rotating disks. The numerical solution of a nonlinear partial differential equation in a rotating porous disk flow near rotating electrodes is achieved using finite difference techniques. It has been reported by Attia et al. [17] that heat transfer in a porous medium can be used to solve a range of differential equations applied to the steady flow over rotating disks.

In many problems of practical interest, many researchers have investigated the fluid flow over a rotating disk because it has various applications in a variety of engineering, industrial, and scientific fields. For example, Zhou et al. [18] presented a mathematical model for Maxwell nanofluid flow with heat transmission over a stretching porous rotating disk. Shafiq et al. [19] investigated the significance of non-linear thermal radiation on Darcy–Forchheimer Casson-water/glycerine nanofluid flow with a uniform magnetic field subject to a rotating disk. Moreover, recently, Alotaibi and Rafique [20] presented a comprehensive analysis of the micro-rotation effect on nanofluid flow generated by a vertical stretching Riga plate. Additional detailed discussions of some recent investigations in the field of nanofluids have been conducted by several researchers and can be found in many related works (see, for example, [21,22,23,24,25,26]).

No analytical solution has been found due to the non-linearities in the reduced differential equation. Mathematica, Maple, and MATLAB are generally employed to solve non-linear equations. Many researchers have adopted approximate analytic methods for non-linear problems. Homotopy perturbation methods (HPMs) [27], homotopy analysis methods (HAMs) [28], and variational iteration methods (VIMs) [29] are among them. Liao [30] describes an analytical technique (HAM) for solving non-linear problems without needing small/significant parameters. Homotopy [31] is a fundamental concept in topology that is the basis for this technique. A porous rotating disk electrode (PRDE) has recently been analyzed by Visuvasam et al. [32]. The mathematical solution will be analytical, whereas more often, this method will be numerical. The method can be applied to various systems and has been described in several publications [33,34,35,36,37,38]. This model can be better understood by reading [39,40,41,42,43] and their references.

To the best of our knowledge, no research has been conducted to investigate the general analytical expressions for the radial, tangential, and axial velocity components. Therefore, the principle aim of this work is to fill that gap by employing the homotopy analysis method, which has been successfully able to interpret the radial, tangential, and axial velocity components for all experimental values. It is expected that the findings of this study will not only be useful in providing excellent information regarding industrial and technical applications, but will also support previously published work.

## 2. Mathematical Formulation of the Problems

**Figure 1:**
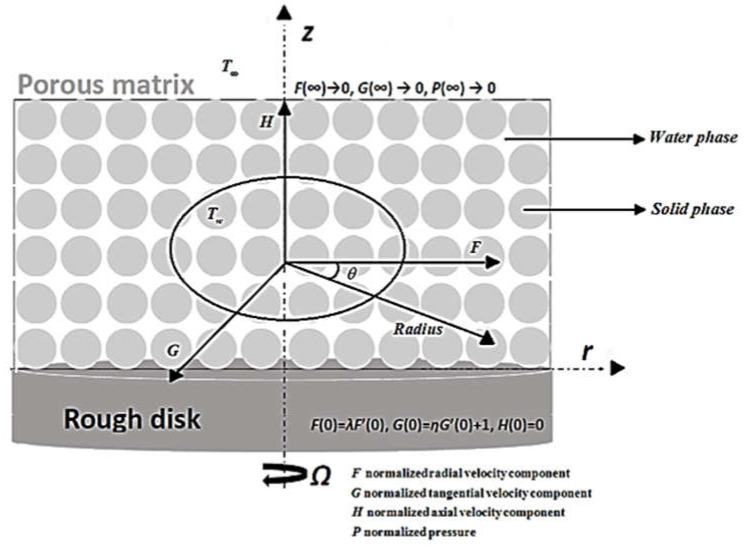
The coordinate system of the Von Kármán flow in a porous medium with its boundary conditions.

We consider an infinite porous rotating disk coinciding with the plane z=0 and with the space z>0 occupied by a viscous incompressible fluid. A steady motion is generated by the rotation of a disk at a constant rotation rate of Ω, which is equivalent to a Darcy model [16,17]. The physical model is outlined in Figure 1. Assuming the flow is laminar, and using the system characteristics described in [33], the continuity and Navier–Stokes equations in cylindrical coordinates are expressed as follows:

(1) $$\frac{u}{r}+\frac{\partial u}{\partial r}+\frac{\partial w}{\partial z}=0$$

(2) $$\rho \left(u\frac{\partial u}{\partial r}-\frac{v^{2}}{r}+w\frac{\partial u}{\partial z}\right)+\frac{\partial p}{\partial r}=\mu \left(\frac{\partial^{2}u}{\partial r^{2}}+\frac{1}{r}\frac{\partial u}{\partial r}-\frac{u}{r^{2}}+\frac{\partial^{2}u}{\partial z^{2}}\right)-\frac{\mu }{K\varepsilon }u$$

(3) $$\rho \left(u\frac{\partial v}{\partial r}-\frac{uv}{r}+w\frac{\partial v}{\partial z}\right)=v\left(\frac{\partial^{2}v}{\partial r^{2}}+\frac{1}{r}\frac{\partial v}{\partial r}-\frac{v}{r^{2}}+\frac{\partial^{2}v}{\partial z^{2}}\right)-\frac{\mu }{K\varepsilon }v$$

(4) $$\rho \left(u\frac{\partial w}{\partial r}+w\frac{\partial w}{\partial z}\right)+\frac{\partial p}{\partial z}=\mu \left(\frac{\partial^{2}w}{\partial r^{2}}+\frac{1}{r}\frac{\partial w}{\partial r}+\frac{\partial^{2}w}{\partial z^{2}}\right)-\frac{\mu }{K\varepsilon }w$$

where u, v, and w are the radial, the angular, and $\gamma =\frac{v}{K\Omega \varepsilon }$ the axial velocity components, respectively. In Equations (1) and (2), r and z are the radial and axial coordinates, respectively, p is the pressure, ρ is the bulk of the fluid, and ν is the kinematic viscosity of the fluid. The partial slip boundary conditions for Equations (1)–(4) are described as follows [44]:

(5) $$u\left(z=0\right)=\lambda_{1}{\bar{\tau }}_{rz}\left(z=0\right),\;v\left(z=0\right)=\lambda_{2}{\bar{\tau }}_{\varphi z}\left(z=0\right)$$

where ${\overset{¯}{\tau }}_{rz}$ and ${\overset{¯}{\tau }}_{\varphi z}$ are the wall shear stresses, and $\lambda_{1}$ and $\lambda_{2}$ are two coefficients. Introducing the following dimensionless [32] variables

(6) $$\xi =\frac{z}{\sqrt{\frac{v}{\Omega }}},\;F=\frac{u}{\Omega r},G=\frac{v}{\Omega r},H=\frac{w}{\sqrt{\Omega v}},P=\frac{p_{\infty }-p}{\rho v\Omega },\;\lambda =\lambda_{1}\sqrt{\frac{\Omega }{v}\mu }$$

Equations (1)–(4) can be rewritten in following the dimensionless form [44]:

(7) $$\frac{dH}{d\zeta }+2F=0$$

(8) $$\frac{d^{2}F}{d\zeta^{2}}-H\frac{dF}{d\zeta }-F^{2}+G^{2}-\gamma F=0$$

(9) $$\frac{d^{2}G}{d\zeta^{2}}-H\frac{dG}{d\zeta }-2FG-\gamma G=0$$

(10) $$\frac{d^{2}H}{d\zeta^{2}}-H\frac{dH}{d\zeta }+\frac{dP}{d\zeta }-\gamma H=0$$

where F,G and H represent the velocity components and γ=v/KΩε is the porosity parameter. The non-dimensional boundary conditions are obtained as follows:

(11) $$F\left(\zeta =0\right)=\lambda F^{\prime }\left(\zeta =0\right),G\left(\zeta =0\right)=\eta G^{\prime }\left(\zeta =0\right)+1,H\left(\zeta =0\right)=0$$

(12) $$\mathrm{and}\;F\left(\zeta \to \infty \right)=0,G\left(\zeta \to \infty \right)=0,P\left(\zeta \to \infty \right)=0$$

## 3. Analytical Expressions of Velocity Components Using the Homotopy Analysis Method

In 1992, Liao [45,46,47,48] first reported HAM as a semi-exact methodology for solving non-linear equations. Some of the most characteristic examples of HAM are found in viscous flows of non-Newtonian fluids [49], KdV-type equations [50], finance [51], and non-linear optimal control problems [52], among others. Therefore, HAM is a useful methodology for solving non-linear problems analytically. In this model, the dimensionless concentrations of radial, tangential, and axial velocity components are approximated analytically using the HAM approach (see Appendix A for more information).

(13) $$F\left(\zeta \right)=\left(A_{2}+A_{5}\right)e^{-\sqrt{\gamma }\zeta }-\left(\frac{e^{-2\sqrt{\gamma }\zeta }}{3\gamma }\right)\left(A_{1}^{2}+h\left(\begin{array}{l} \frac{11}{6}A_{3}A_{1}^{2}\left(h+\frac{4}{11}\right){\left(\sqrt{\gamma }\right)}^{3}+\frac{A_{1}^{2}}{60}\left(\frac{45}{2}A_{2}\gamma e^{-\sqrt{\gamma }\zeta }-A_{1}^{2}e^{-2\sqrt{\gamma }\zeta }\left(h+\frac{4}{3}\right)\right)\\ +\frac{3}{4}A_{2}A_{3}\left(2\gamma^{3}\zeta +{\left(\sqrt{\gamma }\right)}^{5}\right)e^{\sqrt{\gamma }\zeta }+\gamma^{2}\left(A_{1}^{2}A_{3}h\zeta -2A_{3}A_{4}-A_{2}^{2}\right) \end{array}\right)\right)$$

(14) $$G\left(\zeta \right)=\left(A_{1}+A_{4}\right)e^{-\sqrt{\gamma }\zeta }+\frac{1}{24}\frac{A_{1}e^{-3\sqrt{\gamma }\zeta }h\left(-12\gamma^{5/2}\zeta e^{2\sqrt{\gamma }\zeta }+A_{1}^{2}\sqrt{\gamma }-6A_{3}\gamma^{2}e^{2\sqrt{\gamma }\zeta }\right)}{\gamma^{5/2}}$$

(15) $$\begin{array}{l} H\left(\zeta \right)=\frac{2h}{\sqrt{\gamma }e^{\sqrt{\gamma }\zeta }}\left(A_{5}+\frac{A_{2}}{h}\right)+A_{3}+A_{6}-\frac{A_{3}A_{1}^{2}h^{2}}{\gamma^{2}e^{2\sqrt{\gamma }\zeta }}\left(\frac{11}{18}+\frac{2}{9h}-\frac{1}{6\sqrt{\gamma }}-\frac{\zeta }{3}\right)\\ \;\;\;\;\;\;\;\;\;\;\;\;\;\;\;\;\;-h\left(\frac{A_{2}A_{1}^{2}}{12{\left(\sqrt{\gamma }\right)}^{5}e^{3\sqrt{\gamma }\zeta }}-\frac{2A_{1}A_{4}+A_{2}^{2}-\frac{A_{1}^{2}}{h}}{3{\left(\sqrt{\gamma }\right)}^{3}e^{2\sqrt{\gamma }\zeta }}+\frac{A_{3}A_{2}\left(1+\sqrt{\gamma }\zeta \right)}{2\gamma e^{\sqrt{\gamma }\zeta }}-\frac{A_{1}^{4}\left(3h+4\right)}{1080{\left(\sqrt{\gamma }\right)}^{7}e^{4\sqrt{\gamma }\zeta }}\right) \end{array}$$

where ${A_{i}}^{\prime }s$ are constants which are given in Equations (A19), (A26), and (A27).

## 4. Numerical Simulations

**Figure 2:**
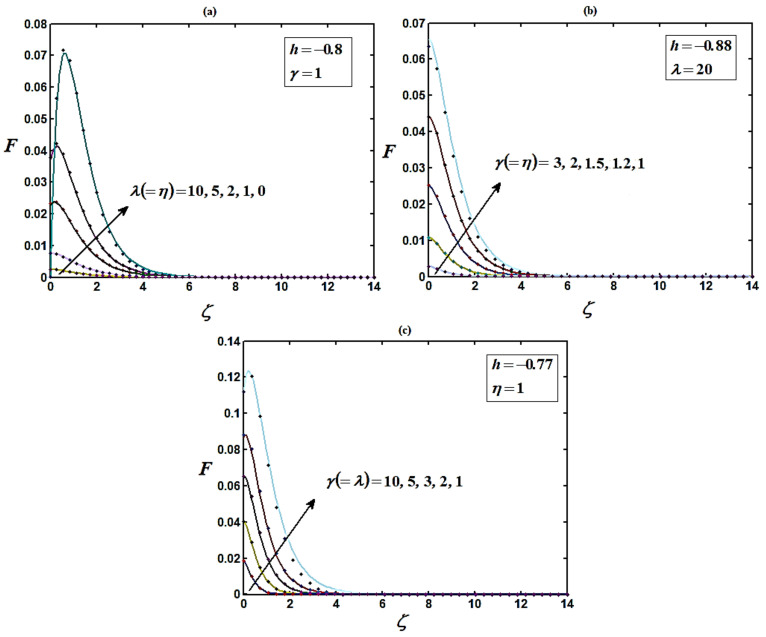
(**a**–**c**) Plots of the normalized radial velocity component F versus normalized distance from the disk ζ using Equation (13) for various values of parameters. Solid lines represent Equation (13) and dotted lines represent the numerical solutions [44].

**Figure 3:**
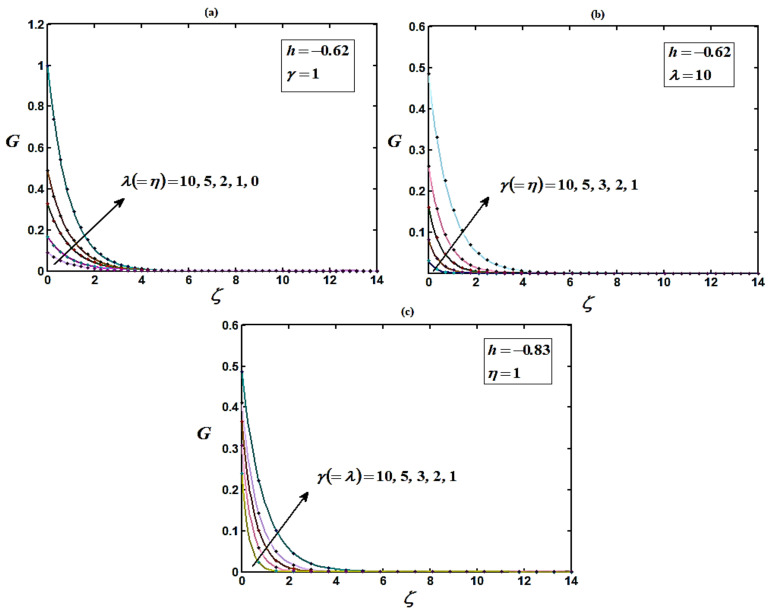
(**a**–**c**) Plots of the normalized tangential velocity component G versus normalized distance from the disk ζ using Equation (14) for various values of parameters. Solid lines represent Equation (14) and dotted lines represent the numerical solutions [44].

**Figure 4:**
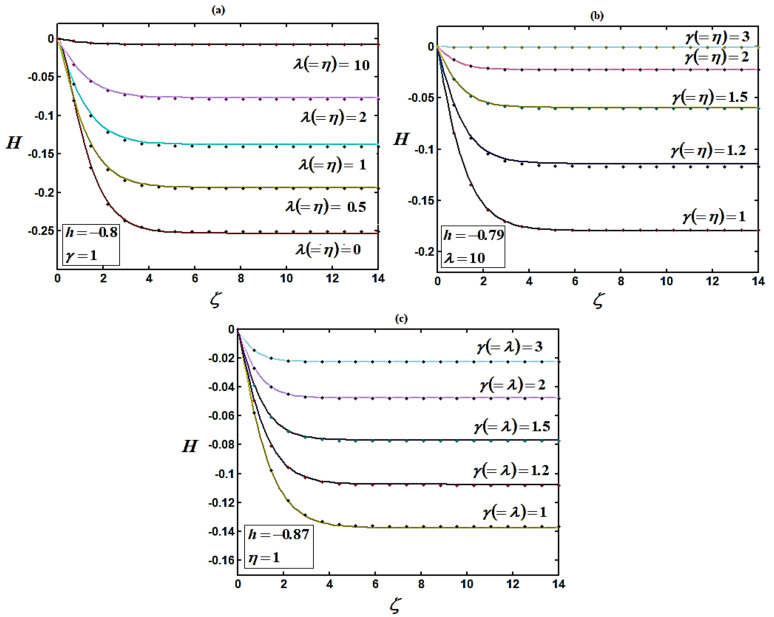
(**a**–**c**) Plots of the normalized axial velocity component H versus normalized distance from the disk ζ using Equation (15) for various values of parameters. Solid lines represent Equation (15) and dotted lines represent the numerical solutions [44].

The precision of the approximate solution obtained with HAM was calculated using symbolic software MATLAB [53] with code bvp4c. Based on a comparison, the analytical results obtained were compatible with the numerical results. Recently, Bikash Sahoo et al. [44] settled the non-linear differential equation system using multiple shooting methods. The homotopy analysis method was compared with the numerical method (MATLAB) and the simulation result provided by Bikash Sahoo et al. [44]. A satisfactory agreement was obtained between both methods, as shown in Figure 2, Figure 3 and Figure 4.

## 5. Discussion

Graphical representation of results is very useful to demonstrate the efficiency and accuracy of the homotopy analysis method for the above problem. This section describes the influence of some interesting parameters on the dimensionless radial, tangential, and axial velocity components. Equations (13)–(15) represent the general new analytical expressions for the dimensionless radial, tangential, and axial velocity components for γ ≥ 1 and for all values of other parameters. It is interesting to compare the impact of each constraint on the velocity components.

The boundary layer slip and porosity parameters are shown in Figure 2, Figure 3 and Figure 4. Figure 2 illustrates that the radial velocity increases proportionally to ζ increase. This pattern is followed until a maximum is reached its asymptotic value (G=0) at ζ=6 and for all values of other parameters. The maximum amount of radial velocity decreases with increasing slip and porosity parameters, which causes the disk to move. Additionally, the radial velocity approaches its asymptotic values at the shortest distance from the disk for large amounts of parameters λ, μ, and γ.

Figure 3 indicates the behavior of the tangential velocity for diverse values of the slip (λ,μ) and porosity (γ) parameters. The tangential velocity reaches its highest value at ζ=0 and decreases monotonically to 0 as ζ=5. For the decreasing quantities of the slip and porosity parameters, axial pace movements in the direction of the disk for its minimum and magnitude also decrease.

Figure 4 indicates that for the increasing values of the slip λ and porosity γ parameters, the steady-state axial velocity increases. Additionally, decreases in porosity parameter leads to reductions in the vertical velocity components at infinity. This is due to the rigidity required by the fluid and the influence of the porosity of the medium. In Figure 4, it is also indicated that the axial velocity is maximum at ζ=0, then decreases slowly and remains constant at ζ ≥ 4. Figure 4 indicates that the vertical velocity component H reaches the full value at a finite distance (ζ ≥ 4) from the disk. The gradual reduction of the peak in the radial component (Figure 1) with decreasing values porosity is reflected in the distributions of the axial velocity components (Figure 4a).

From Figure 2, Figure 3 and Figure 4, it is observed that as the porosity increases the flow becomes more rigid, with the velocity components diminishing over most of the domain (0 ≤ γ ≤ 1). The region close to the disk also contracts in size as porosity grows [44]. It is also observed that the reduction in the axial velocity H is higher than that in the radial velocity F, and much higher than that in the tangential velocity G. This is due to the entire effect of the centrifugal force, which is the source of radial and the vertical motion.

## 6. Differential Sensitivity Analysis of Parameters

**Figure 5:**
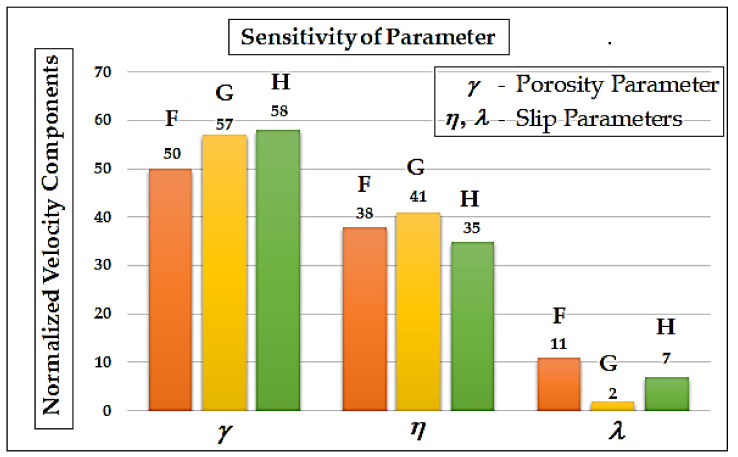
The sensitivity of parameters: percentage changes in normalized velocity components F, G and H  when λ=1, η=1, γ=1 at ζ=1.

Differential sensitivity analysis uses accumulated models that are partially differentiated. The partial derivative of slip parameters (dependent variables) has allowed us to find the partial derivative of porosity parameters (independent variables) at specific fixed experimental values of the parameters (γ = 1, λ=η = 1, and ζ = 1). These parameters make it possible to determine the percentage of change in velocity components. Figure 5 shows the sensitivity analysis of the parameters. The porosity parameter γ has a greater impact on the variation of the velocity components than the slip parameter η, based on Figure 5. The remaining slip parameter λ accounts for only small changes in the velocity components. These results are also confirmed in Figure 5.

## 7. Conclusions

An analysis of Von Kármán swirling flow and heat transfer in porous media has been presented in this paper. The coupled and highly nonlinear differential equations have been solved using the homotopy analysis method. Analysing the velocity component’s behaviour using this analytical result will be valuable. Additionally, the effects and sensitivity analyses of porosity and slip parameters have been discussed. The semi-analytical expressions of the concentration were highly accurate compared to reliable numerical data. Furthermore, they had a greater influence on the heat transfer coefficient than the velocity slip parameter λ. The movement coefficient for maintaining the disk at a constant rotation rate was also enhanced by increasing values of γ. The method described here could easily be applied to the study of other Von Kármán swirling flows and heat transfers in porous media. In our study, we found that boundary layer thickness generally increased with time, but became constant once the steady state was reached; the radial and tangential flow velocities increased both over time and with separation from the disk surface, resulting in accelerated flows.

## Data Availability

Not applicable.

## Abbreviations

***C_m_***	dimensionless moment coefficient (−);
***c_p_***	heat capacity of the fluid at constant pressure (J/kg/K);
***F, G, H***	normalized radial, tangential, and axial velocity components (−);
**K**	Darcy permeability (m^2^);
***Nu***	Nusselt number (−);
***p, P***	pressure (Pa) and normalized pressure (−);
***Pr***	Prandtl number (−);
***q***	heat flux supplied to the disk (W/m^2^);
***r, φ, z***	cylindrical coordinates (m);
***Re***	rotational Reynolds number (−);
***T***	temperature (K);
***u, v, w***	radial, tangential, and axial velocity components (m/s);
***β***	normalized temperature slip factor (−);
***β*_1_**	proportionality constant (−);
***ε***	porosity (−);
***γ***	normalized porosity parameter (−);
***κ***	thermal conductivity of the fluid (W/(m K));
***λ, η***	normalized velocity slip parameters (−);
***µ, ν***	dynamic (Pa.s) and kinematic (m^2^/s) fluid viscosities;
**Ω**	rotation rate of the disk (rad/s);
***ρ***	voluminal mass of the fluid (kg/m^3^);
***ζ***	normalized distance from the disk (−);
***θ***	normalized temperature (−);
***w***	denotes a quantity evaluated at the wall;
∞	denotes a quantity evaluated at infinity;
**‘**	denotes a derived quantity according to the axial direction.

## Appendix A

### Analytical Solution of Equations (7)–(9) Using the Homotopy Analysis Method

In this appendix, we derive the general new analytical solutions of non-linear Equations (7)–(9) using the homotopy analysis method. To find the solutions of Equations (7)–(9), we construct the homotopy as follows:

(A1) $$F^{\prime \prime }-\gamma F+G^{2}-ph\left(-HF^{\prime }-F^{2}\right)=0$$

(A2) $$G^{\prime \prime }-\gamma G-ph\left(-HG^{\prime }-2FG\right)=0$$

(A3) $$H^{\prime }+2F=0$$

where $p\;\in \;\left[0,1\right]$ is the embedding parameter and h ≠ 0 is a non-zero auxiliary parameter.

The approximate solutions of Equations (7)–(9) are:

(A4) $$F=F_{0}+F_{1}p+F_{2}p^{2}+\cdots$$

(A5) $$G=G_{0}+G_{1}p+G_{2}p^{2}+\cdots$$

(A6) $$H=H_{0}+H_{1}p+H_{2}p^{2}+\cdots$$

Substituting Equations (A4)–(A6) in Equations (A1)–(A3) respectively results in:

(A7) $$\begin{array}{l} \left(F_{0}+F_{1}p+\cdots \right)-\gamma \left(F_{0}+F_{1}p+\cdots \right)+{\left(G_{0}+G_{1}p+\cdots \right)}^{2}\\ \;\;\;\;\;\;\;\;\;\;\;+ph\left(\left(H_{0}+H_{1}p+\cdots \right)\left(F_{0}+F_{1}p+\cdots \right)+{\left(F_{0}+F_{1}p+\cdots \right)}^{2}\right)=0 \end{array}$$

(A8) $$\begin{array}{l} \left(G_{0}+G_{1}p+G_{2}p^{2}+\cdots \right)+\gamma \left(G_{0}+G_{1}p+G_{2}p^{2}+\cdots \right)\\ \;\;\;+ph\left(\begin{array}{l} \left(H_{0}+H_{1}p+H_{2}p^{2}+\cdots \right)\left(G_{0}+G_{1}p+G_{2}p^{2}+\cdots \right)\\ +2\left(F_{0}+F_{1}p+F_{2}p^{2}+\cdots \right)\left(G_{0}+G_{1}p+G_{2}p^{2}+\cdots \right) \end{array}\right)=0 \end{array}$$

(A9) $${\left(H_{0}+H_{1}p+H_{2}p^{2}+\cdots \right)}^{\prime }+2\left(F_{0}+F_{1}p+F_{2}p^{2}+\cdots \right)=0$$

Comparing the coefficients of like powers of p in Equation (A7), we obtain:

(A10) $$p^{0}:F_{0}^{\prime \prime }-\gamma F_{0}+G_{0}^{2}=0$$

(A11) $$p^{1}:F_{1}^{\prime \prime }-\gamma F_{1}+2G_{0}G_{1}-h\left(-H_{0}F_{0}^{\prime }-F_{0}^{2}\right)=0$$

Comparing the coefficients of like powers of p in Equation (A8), we have:

(A12) $$p^{0}:G_{0}^{\prime \prime }-\gamma G_{0}=0$$

(A13) $$p^{1}:G_{1}^{\prime \prime }-\gamma G_{1}-h\left(-H_{0}G^{\prime }{}_{0}-2F_{0}G_{0}\right)=0$$

Analyzing the factors of similar powers of p in Equation (A9), we obtain:

(A14) $$p^{0}:H_{0}^{\prime }+2\left(F_{0}\right)=0$$

(A15) $$p^{1}:H_{1}^{\prime }+2\left(F_{1}\right)=0$$

The boundary conditions in Equations (12) and (13) become:

(A16) $$F_{0}\left(0\right)=\lambda F_{0}^{\prime }\left(0\right),\;G_{0}\left(0\right)=\eta G_{0}^{\prime }\left(0\right)+1,\;H_{0}\left(0\right)=0\;F_{i}\left(0\right)=\lambda {F_{i}^{\prime }}^{\prime }\left(0\right),G_{i}\left(0\right)=\eta G_{i}^{\prime }\left(0\right),H_{i}\left(0\right)=0,F_{i-1}\left(\infty \right)=G_{i-1}\left(\infty \right)=0\forall i=1,2,3\ldots$$

Solving Equations (A10) and (A4) and using the boundary conditions (A16), we find:

(A17) $$F_{0}\left(\zeta \right)=A_{2}e^{-\sqrt{\gamma }\zeta }-\frac{1}{3}\frac{A_{1}^{2}e^{-2\sqrt{\gamma }\zeta }}{\gamma }$$

(A18) $$F_{1}\left(\zeta \right)=A_{5}e^{-\sqrt{\gamma }\zeta }-\frac{e^{-4\sqrt{\gamma }\zeta }h}{3\gamma }\left(\begin{array}{l} \left(\frac{11}{6}A_{3}A_{1}^{2}\left(h+\frac{4}{11}\right)\gamma^{3/2}+\gamma^{2}\left(A_{1}^{2}A_{3}h\zeta -2A_{3}A_{4}-A_{2}^{2}\right)\right)e^{2\sqrt{\gamma }\zeta }\\ +\frac{3}{4}A_{2}A_{3}\left(2\gamma^{3}\zeta +\gamma^{5/2}\right)e^{3\sqrt{\gamma }\zeta }-\frac{1}{60}\left(\frac{-45}{2}A_{2}\gamma e^{\sqrt{\gamma }\zeta }+A_{1}^{2}\left(h+\frac{4}{3}\right)\right)A_{1}^{2} \end{array}\right)$$

where

(A19) $$A_{1}=\frac{1}{\sqrt{\gamma }\eta +1},\;A_{2}=\frac{A_{1}^{2}\left(2\sqrt{\gamma }\lambda +1\right)}{3\gamma \left(\sqrt{\gamma }\lambda +1\right)},\;A_{3}=\frac{A_{1}^{2}-6A_{2}\gamma }{3{\sqrt{\gamma }}^{3}},\;A_{4}=-\frac{1}{24}\frac{A_{1}h\left(6A_{3}\eta \gamma^{4}+3A_{1}^{2}\eta \sqrt{\gamma }+A_{1}^{2}-6A_{3}\sqrt{\gamma^{3}}\right)}{\gamma^{2}\left(\eta \sqrt{\gamma }+1\right)}$$

Considering two iterations, we obtain

(A20) $$F\left(\zeta \right)\approx F_{0}\left(\zeta \right)+F_{1}\left(\zeta \right)$$

After putting Equations (A17) and (A18) in (A20), the final results can be described as Equation (13) in the text. Similarly, solving Equations (A12) and (A13) and using the boundary conditions (A16), we obtain:

(A21) $$G_{0}\left(\zeta \right)=A_{1}e^{-\sqrt{\gamma }\zeta }$$

(A22) $$G_{1}\left(\zeta \right)=A_{4}e^{-\sqrt{\gamma }\zeta }+\frac{1}{24}\frac{A_{1}e^{-3\sqrt{\gamma }\zeta }h\left(-12\gamma^{5/2}\zeta e^{2\sqrt{\gamma }\zeta }+A_{1}^{2}\sqrt{\gamma }-6A_{3}\gamma^{2}e^{2\sqrt{\gamma }\zeta }\right)}{\gamma^{5/2}}$$

According to the HAM, we can obtain the solutions of Equation (9) as follows:

(A23) $$G\left(\zeta \right)\approx G_{0}\left(\zeta \right)+G_{1}\left(\zeta \right)$$

After substituting Equations (A21) and (A22) in (A23), the final results can be described as Equation (14) in the text. Using the same procedure to solve Equations (A14) and (A15), we find:

(A24) $$H_{0}\left(\zeta \right)=\frac{2A_{2}e^{-\sqrt{\gamma }\zeta }}{\sqrt{\gamma }}-\frac{A_{1}^{2}e^{-2\sqrt{\gamma }\zeta }}{3\gamma^{3/2}}+A_{3}$$

(A25) $$\begin{array}{l} H_{1}\left(\zeta \right)=\frac{A_{3}A_{1}^{2}h^{2}}{\gamma^{2}e^{2\sqrt{\gamma }\zeta }}\left(\frac{11}{18}+\frac{2}{9h}-\frac{1}{6\sqrt{\gamma }}-\frac{\zeta }{3}\right)+\frac{2A_{5}h}{\sqrt{\gamma }e^{\sqrt{\gamma }\zeta }}\\ -h\left(+\frac{A_{2}A_{1}^{2}}{12{\left(\sqrt{\gamma }\right)}^{5}e^{3\sqrt{\gamma }\zeta }}-\frac{\left(2A_{1}A_{4}+A_{2}^{2}\right)}{3r^{3}e^{2\sqrt{\gamma }\zeta }}+A_{3}A_{2}\left(\frac{1+\sqrt{\gamma }\zeta }{2\gamma e^{\sqrt{\gamma }\zeta }}\right)-\frac{A_{1}^{4}\left(3h+4\right)}{1080r^{7}e^{4\sqrt{\gamma }\zeta }}\right)+A_{6} \end{array}$$

where

(A26) $$A_{5}=\frac{h}{360\left(\lambda \sqrt{\gamma }+1\right)}\left(\begin{array}{l} \frac{320}{\gamma }\left(A_{1}^{2}A_{3}\lambda \left(h+\frac{1}{2}\right)-\frac{3}{4}A_{1}A_{4}-\frac{3}{8}A_{2}^{2}\right)+30\lambda \left(2A_{1}A_{4}+A_{2}^{2}-\frac{3}{8\lambda }A_{3}A_{2}\right)\\ -\frac{8}{\gamma }\left(\left(\frac{1}{4}+\lambda \right)\left(h+\frac{4}{3}\right)\frac{A_{1}^{4}}{\gamma^{2}}-\frac{55}{2}\left(\frac{27}{44}A_{2}\lambda +A_{3}\left(h+\frac{4}{11}\right)\right)\frac{A_{1}^{2}}{\gamma }\right)+45\left(\frac{A_{1}^{2}}{{\left(\sqrt{\gamma }\right)}^{7}}-2A_{3}\lambda \right)A_{2} \end{array}\right)$$

(A27) $$A_{6}=\frac{h}{\left(\lambda \sqrt{\gamma }+1\right)}\left(\begin{array}{l} \left(A_{1}^{2}A_{3}\lambda \left(h+\frac{2}{3}\right)-\frac{2}{3}A_{1}A_{4}-\frac{1}{3}A_{2}^{2}\right)\gamma +\left(\frac{A_{1}^{2}}{6r}-2A_{3}\lambda {\left(\sqrt{\gamma }\right)}^{3}\right)A_{2}-\frac{A_{1}^{4}}{24}\left(h+\frac{4}{3}\right)\left(\frac{1}{5{\left(\sqrt{\gamma }\right)}^{3}}+\frac{\lambda }{\gamma }\right)\\ +\frac{4A_{1}^{2}}{9}\left(\frac{3}{2}A_{2}\lambda +A_{3}\left(h+\frac{1}{2}\right)\right)-\lambda \left(2A_{1}A_{4}+A_{2}^{2}+\frac{A_{3}A_{2}}{\lambda }\right)\gamma \end{array}\right)$$

Additionally,

(A28) $$H\left(\zeta \right)\approx H_{0}\left(\zeta \right)+H_{1}\left(\zeta \right)$$

Substituting Equations (A24) and (A25) in (A28), the final results can be described as Equation (15) in the text.
